# Broadband THz Absorption of Microbolometer Array Integrated with Split-Ring Resonators

**DOI:** 10.1186/s11671-020-03454-2

**Published:** 2020-12-03

**Authors:** Shuming Fan, Jun Gou, Qingchen Niu, Zheyuan Xie, Jun Wang

**Affiliations:** 1 School of Optoelectronic Science and Engineering, University of Electronic Science and Technology of China, Chengdu, 610054 China; 2 State Key Laboratory of Electronic Thin Films and Integrated Devices, University of Electronic Science and Technology of China, Chengdu, 610054 China

**Keywords:** Thz, Microbolometer, Split-ring, Absorption, Broadband

## Abstract

In this paper, a periodic structure based on metallic split-ring resonators is integrated into micro-bridge structures of THz microbolometer array to achieve high THz wave absorption in a wide frequency range. With a small unit size of 35 μm × 35 μm, the effect of split-ring structure on THz wave absorption characteristics of the multilayer structure array is studied to manipulate the resonance absorption frequencies. The absorption bandwidth is effectively increased by integrating a combined structure of split-ring and metallic disk. Broadband THz absorption is formed by coupling the absorption peaks of different structures. The periodic structure of dual-ring combined with a metallic disk provides a broadband THz wave absorption in the range of 4–7 THz. The highest absorption in the band reaches 90% and the lowest absorption is higher than 40%. The designed structure is process-compatible and easy to implement for small-pixel THz microbolometers with high absorption in a wide spectrum range. The research provides a scheme for broadband THz sensing and real-time imaging at room temperature.

## Introduction

Terahertz (THz) wave with a wavelength of 30 μm to 3 mm is a very important but rarely explored portion of the electromagnetic spectrum. Applications of THz technology include security screening [1, 2], medicine [3, 4], communication [5, 6] and astronomy [7]. THz technology has been undergoing tremendous progress in recent years due to the development of sources and devices for generation and detection of THz wave [8, 9]. THz detectors are mainly based on photoelectric effect and thermal effect. Photon detectors such as superconducting bolometers can be used for high-sensitivity and high-speed detecting [10, 11]; however, it needs to be cooled down to extremely low temperature. Thermal bolometer detectors that absorb THz wave and cause temperature change of thermal sensitive film can be operated at room temperature and have great advantages in large-scale array integration, simple configuration and low cost [12–14]. THz microbolometer array is composed of pixels with micro-bridge structure, which is developed from mature infrared (IR) microbolometer technology with the same thermal conversion mechanism equipped with a THz source. A critical drawback of conventional micro-bridge structure is its poor absorption of THz wave, which causes low sensitivity. Some improvements have been made on micro-bridge structure for enhanced THz absorption, including integrating an impedance matching metallic thin film and an antenna tuned to the target frequency [15–18]. However, metallic thin film exhibits limited absorption (≤ 50%), while antenna-coupled micro-bridge structure generally has a narrow absorption peak of THz wave. In order to achieve high THz absorption in a wide spectrum range, a thin dielectric layer and a thin metal layer can be added on the top surface of a conventional three-layer absorber [19]. The phase-coupled method and strong coupling response can also improve the absorption bandwidth or realize multiband absorption [20–23]. However, most of the structures cannot be integrated in the small pixels with micro-bridge structures of THz microbolometer array without sacrificing the thermal and mechanical properties.

Split-ring resonator is a widely studied structure to manipulate electromagnetic waves by exciting surface plasmon trapped in the periodic structure [24, 25]. In this paper, with the purpose of enhancing the absorption of THz microbolometer array, metallic split-ring with four openings is integrated into the micro-bridge structure with a small size of 35 μm × 35 μm. To increase the absorption bandwidth, periodic structures of split-ring resonators combined with another split-ring and a metallic disk are studied. Broadband THz absorption is achieved by coupling the absorption peaks of different structures. The dual-ring structure combined with an aluminum (Al) disk provides a broadband THz wave absorption in the range of 4–7 THz with the highest absorption of 90% and the lowest absorption higher than 40%. Phase-coupled method and strong coupling response can also improve the bandwidth or realization of multiband absorption.

## Results and Discussion

**Fig. 1:**
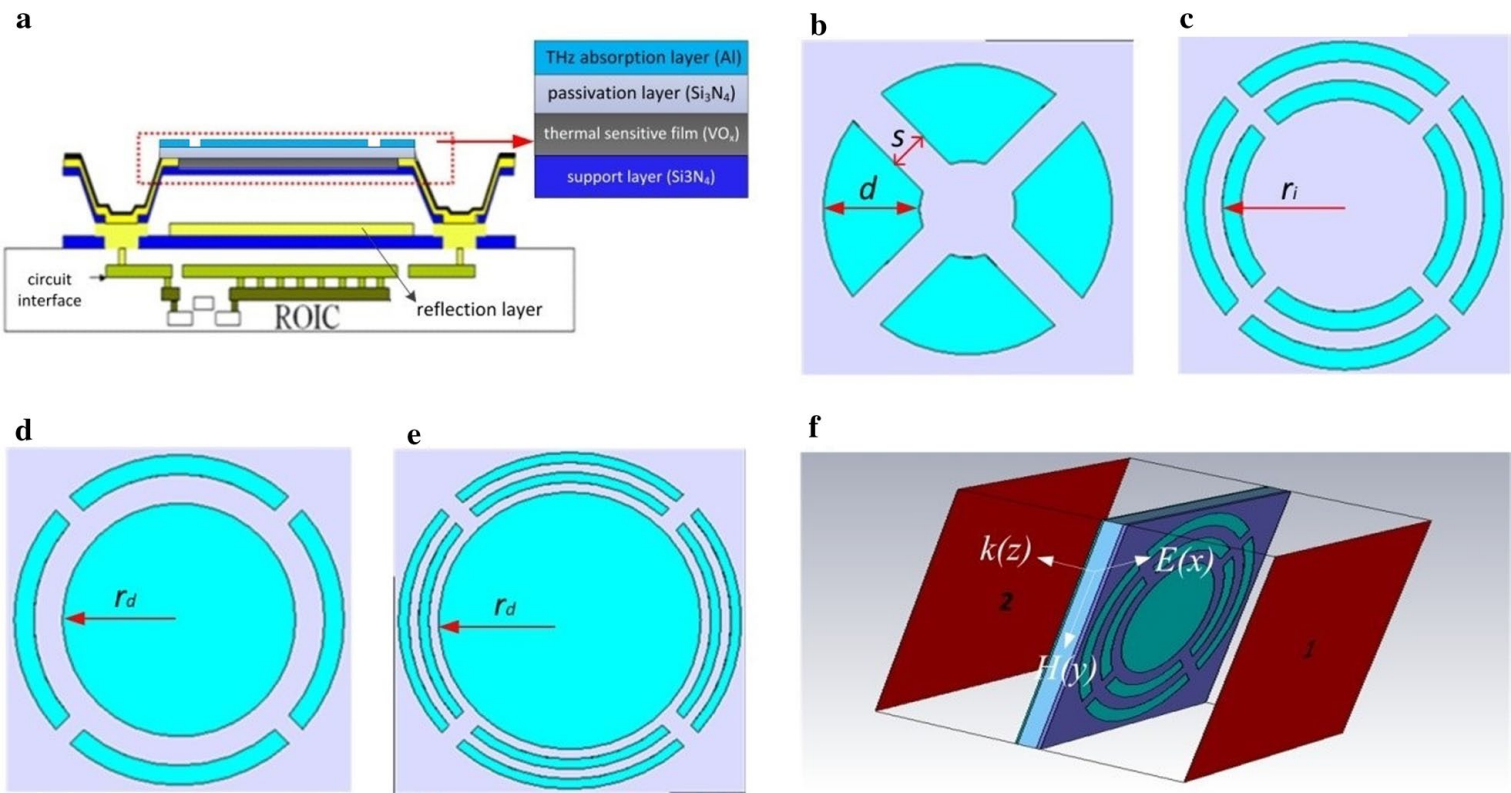
Design of micro-bridge structure coupled with split-ring resonators. **a** Sectional view of micro-bridge structure. **b** Split-ring with four openings. **c** Dual-ring structure. **d** A split-ring combined with an Al disk. **e** Dual-ring structure combined with an Al disk. **f** A single unit cell of THz microbolometer array illuminated by vertical incident light

THz microbolometer array is composed of many micro-bridge structure pixels in two-dimensional repeated arrangement on the focal plane. Each pixel measures THz radiation independently. The micro-bridge structure is shown in Fig. 1a, which consists of a sensitive multilayered film and two legs supporting the film. The multilayered film includes a 250-nm support layer (silicon nitride, Si_3_N_4_), a 60-nm thermal sensitive film (vanadium oxide, VO_*x*_), a 150-nm passivation layer (Si_3_N_4_) and a THz wave absorption layer (Al) from bottom to top. The legs are used for mechanical supporting, electrical and thermal channels. VO_*x*_ film is connected through the legs with the electrodes of readout circuit (ROIC) integrated into silicon (Si) substrate. THz wave absorbed by the absorption layer causes a temperature change of the multilayered film and a resistance change of VO_x_ film which is detected by ROIC. A 2-μm-high cavity for thermal insulation is formed between the reflective layer (Al) with a thickness of 400 nm on Si substrate and the sensitive multilayered film. In this paper, split-ring with four openings, as shown in Fig. 1b, is integrated into micro-bridge structure as the THz absorption layer. In order to increase THz absorption bandwidth, dual-ring structure as shown in Fig. 1c, a split-ring combined with an Al disk as shown in Fig. 1d and dual-ring structure combined with an Al disk as shown in Fig. 1e are also studied.

**Fig. 2:**
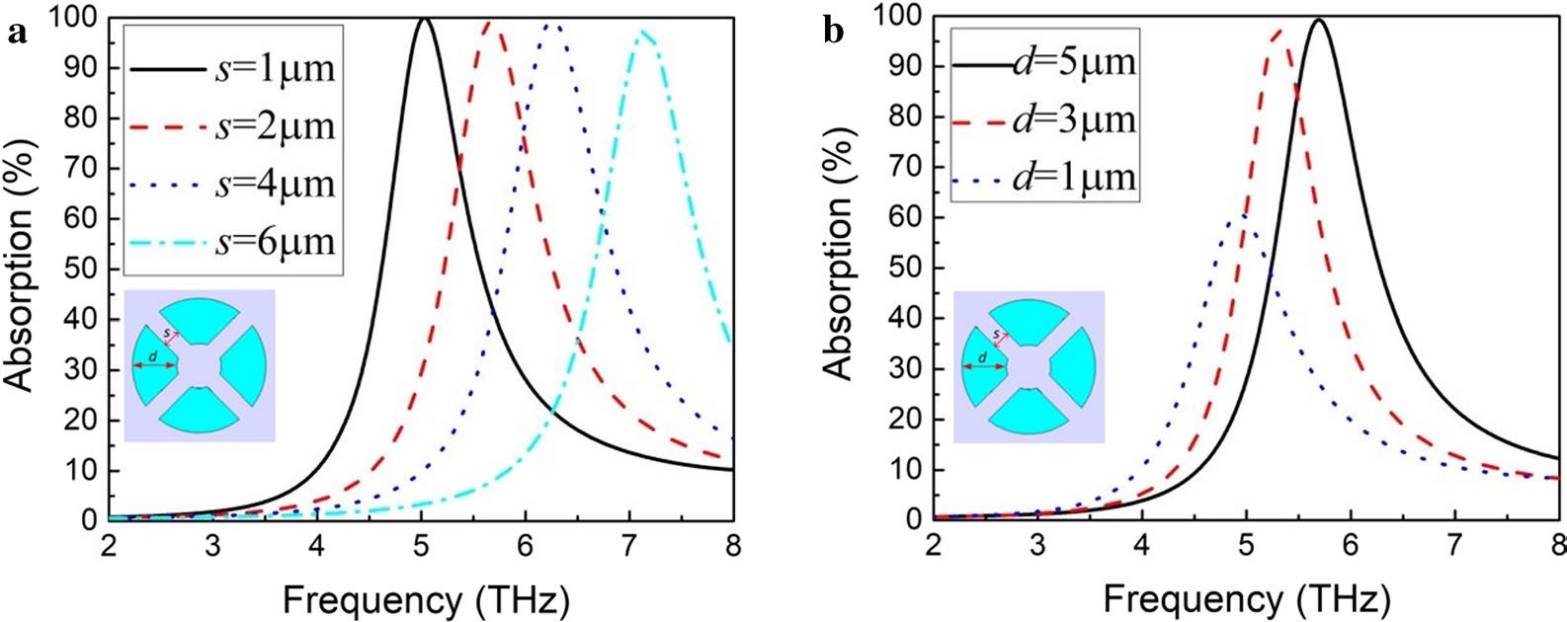
**a** THz wave absorption of periodic split-ring structures with different opening widths (*s*). The split-rings have an outer radius of 15 μm, an inner radius of 10 μm and a thickness of 10 nm. **b** THz wave absorption of periodic split-ring structures with different ring widths (*d*). The split-rings have an outer radius of 15 μm, an opening width of 2 μm and a thickness of 10 nm

Figure 2a shows THz wave absorption of periodic split-ring structures with different opening widths (*s*). The split-rings have an outer radius of 15 μm, an inner radius of 10 μm and a thickness of 10 nm. When the opening width of split-rings is 1 μm, 2 μm, 4 μm and 6 μm, the resonance absorption frequency is 5 THz, 5.7 THz, 6.2 THz and 7.1 THz, respectively. The peak absorption of each structure is about 100%. With the increase of opening width, the resonance absorption frequency increases. The openings of the split-ring can be regarded as equivalent capacitance (*C*) while the metallic ring part of the split-ring can be regarded as equivalent inductance (*L*) and the resonance frequency ($\omega$) can be expressed as $\omega =\frac{1}{\sqrt{LC}}$. The increase of the opening width results in the reduction of equivalent capacitance and the increase of resonance frequency. Therefore, high resonance absorption at a lower frequency can be achieved with a smaller opening width of the split-ring. Figure 2b shows THz wave absorption of periodic split-ring structures with different ring widths (*d*). The split-rings have an outer radius of 15 μm, an opening width of 2 μm and a thickness of 10 nm. It can be seen that with the decrease of ring width, the resonance absorption frequency and the peak absorption decrease. The peak absorption reaches 100% at 5.7 THz and 97% at 5.3 THz with a ring width of 5 μm and 3 μm, respectively. When the ring width is 1 μm, the resonance absorption frequency is 5 THz and the peak absorption decreases to 60%. The decrease of resonance absorption frequency is attributed to the increase of equivalent inductance as the ring width decreases.

**Fig. 3:**
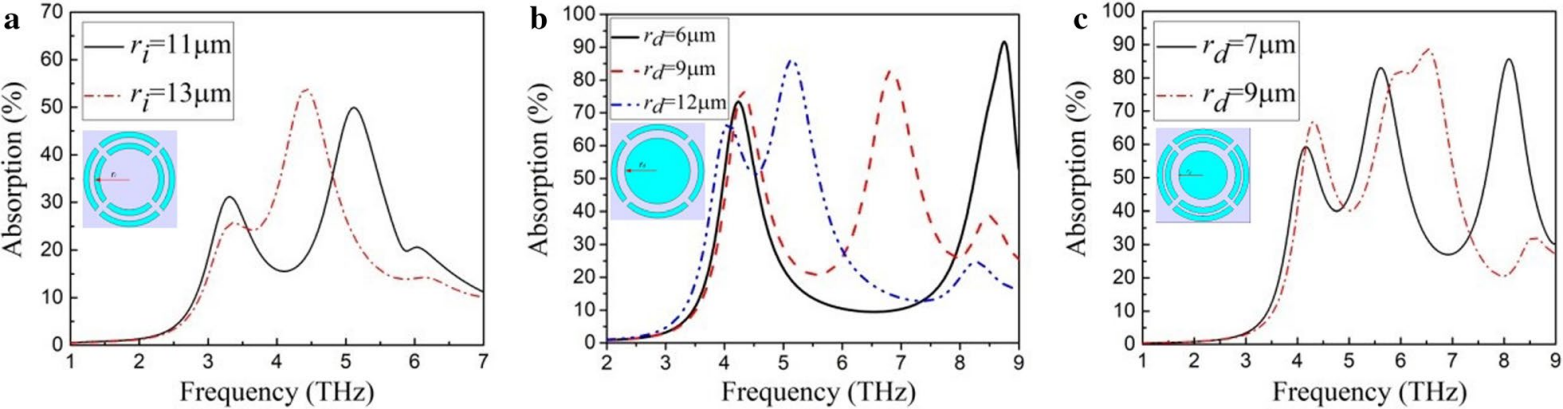
**a** THz wave absorption of periodic dual-ring structures with different outer radii of the inner split-ring (*r*_i_). The dual-ring structures have an opening width of 2 μm and a thickness of 10 nm. The outer radius of the outer split-ring is 17 μm and the width of both split-rings is 2 μm. **b** THz wave absorption of periodic structures of a combination of a split-ring and an Al disk with different radii of the disk (*r*_d_). The periodic structures have a thickness of 10 nm. The split-ring has an outer radius of 17 μm, a ring width of 2 μm and an opening width of 2 μm. **c** THz wave absorption of periodic structures of a combination of two split-rings and an Al disk with different radii of the disk (*r*_d_). The periodic structures have a thickness of 10 nm. The two split-rings have a ring width of 2 μm, an opening width of 2 μm and an outer radius of 17 μm and 14 μm, respectively

The periodic split-ring structure can provide high THz wave absorption at the resonance frequency. However, the absorption peak is narrow. In order to increase the absorption bandwidth, periodic structures of several different combinations of split-ring and Al disk are integrated into micro-bridge structure arrays. Figure 3a shows THz wave absorption of periodic dual-ring structures with different outer radius of the inner split-ring (*r*_i_). The dual-ring structure has an opening width of 2 μm and a thickness of 10 nm. The outer radius of the outer split-ring is 17 μm and the width of both split-rings is 2 μm. The dual-ring structures have two absorption peaks. As the outer radius of the inner split-ring increases from 11 to 13 μm, one resonance absorption frequency keeps unchanged at 3.3 THz while the other resonance absorption frequency decreases from 5.1 to 4.3 THz. The absorption peaks at lower frequency and higher frequency are, respectively, contributed by the outer split-ring and the inner split-ring. As the two split-rings get closer, the two absorption peaks are coupled with each other and form a wider absorption band. However, this structure exhibits a relatively low absorption of 25–55% in the absorption band of 3.2–5.2 THz.

THz wave absorption of periodic structures of a combination of a split-ring and an Al disk with different radii of the disk (*r*_d_) is shown in Fig. 3b. The periodic structures have a thickness of 10 nm. The split-ring has an outer radius of 17 μm, a ring width of 2 μm and an opening width of 2 μm. The periodic structures have two absorption peaks. One of the absorption peaks is located near 4.3 THz, which does not change with the radius of Al disk. With the increase of the radius of the disk from 6 to 12 μm, the other absorption peak at higher frequency moves toward lower-frequency direction and the change of peak absorption is not significant. The absorption peak near 4.3 THz is contributed by the split-ring while the absorption peak at higher frequency moving with the change of disk structure is contributed by the Al disk. When the radius of the disk is 12 μm, a broadband absorption is obtained with a width of about 2 THz. Figure 3c shows THz wave absorption of periodic structures of a combination of two split-rings and an Al disk with different radii of the disk (*r*_*d*_). The periodic structures have a thickness of 10 nm. The two split-rings have a ring width of 2 μm, an opening width of 2 μm and an outer radius of 17 μm and 14 μm, respectively. The resonance absorption frequency is around 4.2 THz for the outer split-ring and between 5.5 and 6 THz for the inner split-ring. When the radius of Al disk is 7 μm, the resonance absorption peak is at 8.2 THz. When the radius of the disk is 9 μm, its absorption peak moves to 6.5 THz and couples with the absorption peak of the inner split-ring. The periodic structure of a combination of two split-rings and an Al disk provides a broadband absorption in 4–7 THz. The highest absorption in the band reaches 90% and the lowest absorption is higher than 40%.

**Fig. 4:**
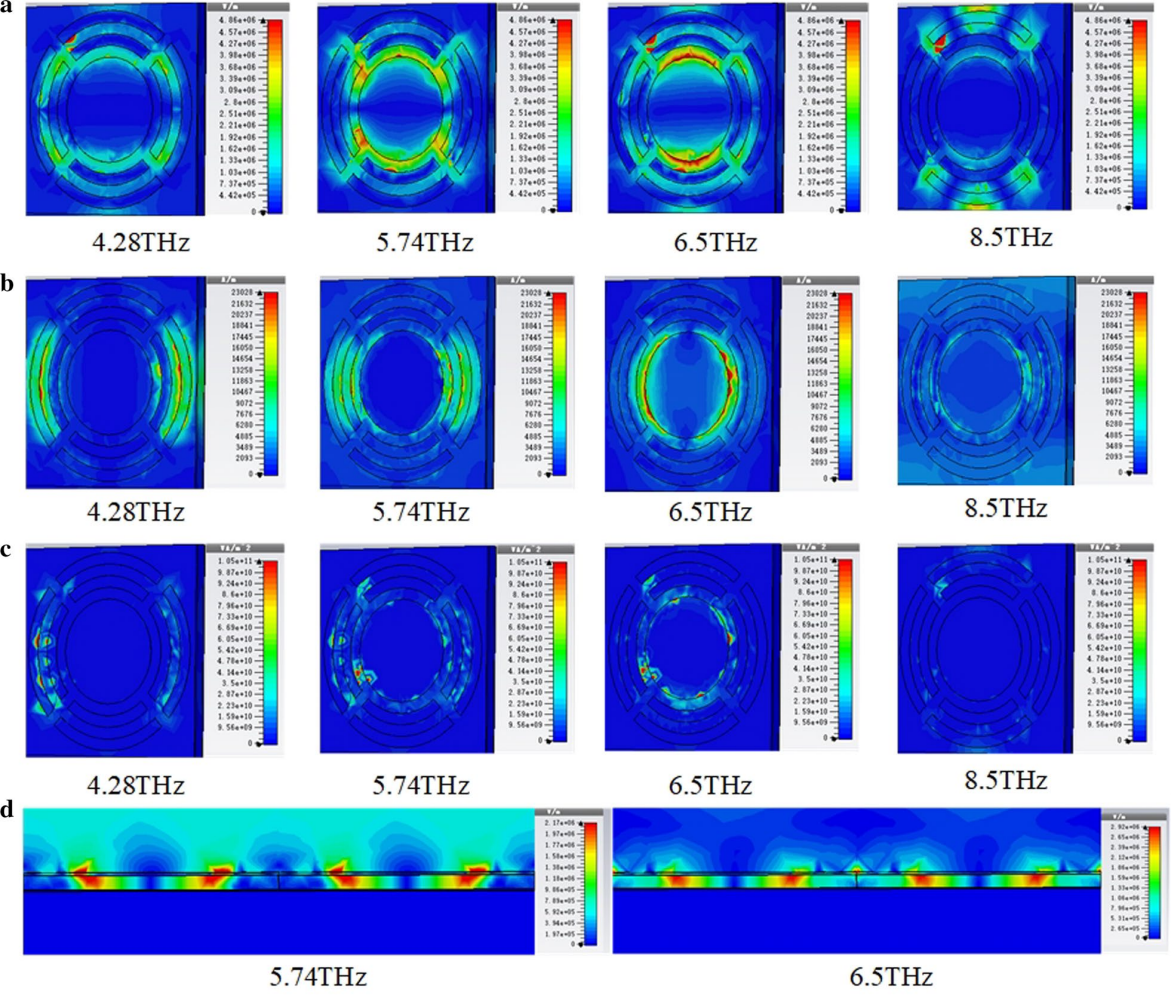
Top view of electric field density distribution (**a**), magnetic field density distribution (**b**), power loss (**c**) and sectional view of electric field density distribution (**d**) in the periodic structure of two split-rings combined with an Al disk at different resonance absorption frequencies. The periodic structure has a thickness of 10 nm. The two split-rings have a ring width of 2 μm, an opening width of 2 μm and an outer radius of 17 μm and 14 μm, respectively. The Al disk has a radius of 9 μm

Figure 4 shows the distribution of electric field energy density, magnetic field energy density and power loss in the periodic dual-ring structure combined with an Al disk at different resonance absorption frequencies. The periodic structure has a thickness of 10 nm. The two split-rings have a ring width of 2 μm, an opening width of 2 μm and an outer radius of 17 μm and 14 μm, respectively. The Al disk has a radius of 9 μm. As shown in Fig. 3c, this periodic structure has four absorption peaks at the frequencies of 4.28 THz, 5.74 THz, 6.5 THz and 8.5 THz. The distribution of electric field energy density, magnetic field energy density and power loss at the four resonance absorption frequencies shows the main absorption areas of THz wave in the structure. It can be seen that the outer split-ring, the inner split-ring and the disk contribute mainly to the resonance absorption at 4.28 THz, 5.74 THz and 6.5 THz, respectively. This supports the previous analysis of the absorption peaks. The low absorption peak at 8.5 THz is attributed to the coupling of periodic structures. Figure 4d shows the sectional view of electric field density distribution in the periodic dual-ring structure combined with an Al disk at resonance absorption frequencies of 5.74 THz and 6.5 THz. Strong electric field can be observed at the metal layer and dielectric layer. The absorption is mainly attributed to the ohmic loss at the metal layer and dielectric loss at the dielectric layer. Most of the absorption occurs on the support layer and can be transformed to temperature rise of the VO_x_ thin film.

**Fig. 5:**
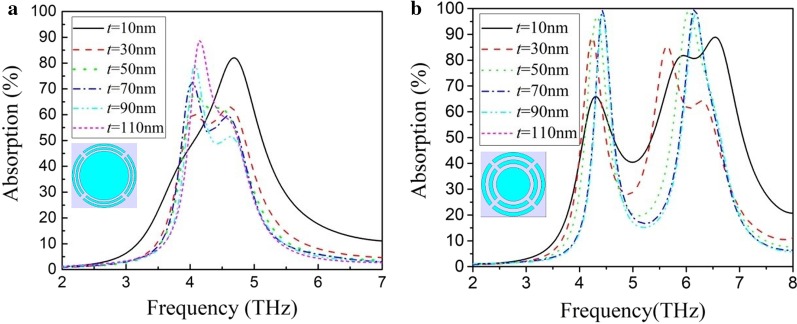
THz wave absorption of periodic structures of a combination of two split-rings and an Al disk with different thicknesses (*t*). **a** Two split-rings have a ring width of 1 μm, an opening width of 2 μm and an outer radius of 17 μm and 15 μm, respectively. The Al disk has a radius of 13 μm. **b** Two split-rings have a ring width of 2 μm, an opening width of 2 μm and an outer radius of 17 μm and 13 μm, respectively. The Al disk has a radius of 9 μm

THz wave absorption of periodic structures of a combination of two split-rings and an Al disk with different thicknesses (*t*) is shown in Fig. 5. In the periodic structures in Fig. 5a, the two split-rings have a ring width of 1 μm, an opening width of 2 μm and an outer radius of 17 μm and 15 μm, respectively. The Al disk has a radius of 13 μm. The distance between adjacent structures is 1 μm. The absorption peaks of different structures are coupled together and form a wide absorption band. As the thickness of the absorption layer increases, the absorption bandwidth becomes narrower. However, when the thickness is greater than 30 nm, the periodic structure's absorption characteristic does not change significantly, showing a relatively stable absorption. In the periodic structures in Fig. 5b, the two split-rings have a ring width of 2 μm, an opening width of 2 μm and an outer radius of 17 μm and 13 μm, respectively. The Al disk has a radius of 9 μm. The distance between adjacent structures is 2 μm. When the thickness of the absorption layer is 10 nm, this periodic structure provides a broadband absorption in 4–7 THz with a THz wave absorption of 40–90% in the band. As the thickness increases, the absorption band gradually becomes two independent absorption peaks. Although the peak absorption is very high, it is difficult to form a wide absorption band of THz wave.

**Fig. 6:**
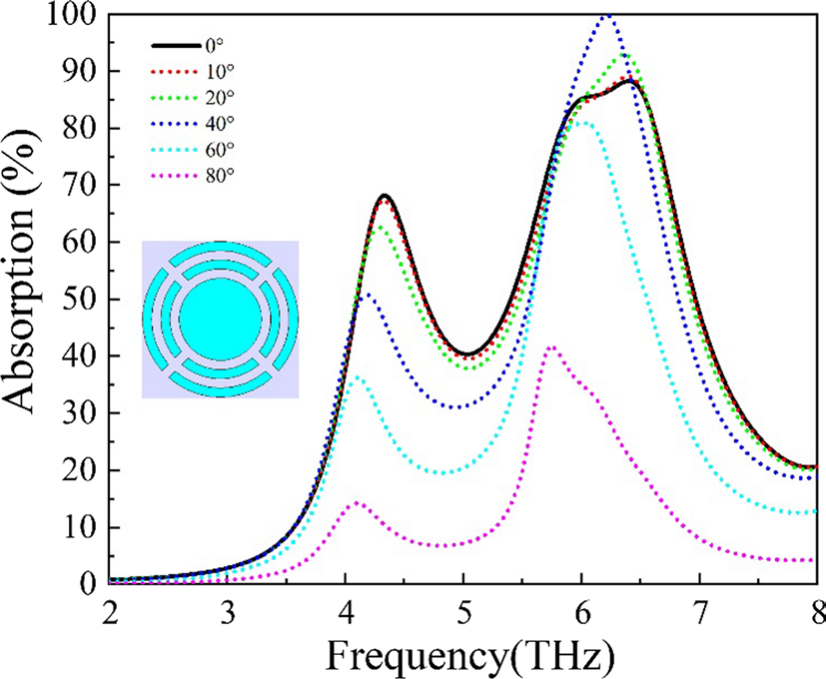
THz wave absorption of periodic structures of a combination of two split-rings and an Al disk with different incident angles. The two split-rings have a ring width of 2 μm, an opening width of 2 μm and an outer radius of 17 μm and 13 μm, respectively. The Al disk has a radius of 9 μm and a thickness of 10 nm

In order to investigate the absorption property under illumination of oblique incidence, THz wave absorption of periodic structures of a combination of two split-rings and an Al disk with different incident angles of 0° (normal incidence), 10°, 20°, 40°, 60° and 80° are simulated and shown in Fig. 6. In the periodic structures, the two split-rings have a ring width of 2 μm, an opening width of 2 μm and an outer radius of 17 μm and 13 μm, respectively. The Al disk has a radius of 9 μm and a thickness of 10 nm. The distance between adjacent structures is 2 μm. As the incident angle increases, the two peak absorption frequencies move toward lower frequency direction slightly. When the incident angle is less than 30°, the change of peak absorption rate is not significant. However, the absorption strength will decrease significantly when the incident angle is greater than 40°.

## Conclusions

Periodic structures based on Al split-ring resonators in micro-bridge structure array with a unit size of 35 μm × 35 μm are studied with the purpose of improving THz wave absorption and increase the absorption bandwidth of THz microbolometers. The resonance absorption frequency of split-ring resonators is determined by the opening width and the width of the ring. Periodic structures with a combination of split-rings and Al disk are integrated into micro-bridge structure arrays. By coupling the absorption peaks of different structures, the absorption bandwidth is effectively increased. High THz wave absorption in the frequency range of 4–7 THz with absorption of 40–90% is achieved by the periodic dual-ring structure combined with a disk. The structure meets the requirements of THz microbolometers for small pixel size, high absorption and wide spectrum response.

## Methods

We performed finite-element numerical simulations using CST Microwave Studio 2016. We simulated a single cubic unit cell of THz microbolometer array with a size of 35 μm × 35 μm, as shown in Fig. 1f. The wave vector *k* propagated through the *z* direction with perfect electric field in *x–z* plane and perfect magnetic field in *y–z* plane. We set the input and output ports on the top and bottom faces of the cubic unit cell, which are indicated as port “1” and port “2,” respectively. The simulation produced frequency-dependent complex *S* parameters, from which we obtained the reflectance *R* =|*S*_*11*_|^2^ at port “1” and transmittance *T* =|S_21_|^2^ at port “2” with periodic boundary conditions (PBC) along the *x* and *y* directions. The absorption of the periodic structure was calculated via *A* = 1 −|*S*_*11*_|^2^ −|*S*_*21*_|^2^. For the structures proposed in Fig. 1b–e, the Al absorption layer and the reflection layer were modeled using Drude model with a plasma frequency of ${\omega }_{p}=$ 92,700 cm^−1^ and a scattering frequency of ${\omega }_{\tau }=$ 408 cm^−1^ [26]. The support and passivation layer with a total thickness of 400 nm was modeled as optical Si_3_N_4_ film with a dispersion permittivity of the second-order model (fit) in CST and a permeability of 1. The cavity was modeled with a permittivity of 1 and a permeability of 0 S/m.

## Data Availability

All data supporting the conclusions of this article are included within the article.

## Abbreviations

THz: Terahertz
IR: Infrared
Al: Aluminum
Si_3_N_4_: Silicon nitride
VO_x_: Vanadium oxide
ROIC: Readout integrated circuit
Si: Silicon
PBC: Periodic boundary conditions
